# The MARAS dataset, vegetation and soil characteristics of dryland rangelands across Patagonia

**DOI:** 10.1038/s41597-020-00658-0

**Published:** 2020-10-05

**Authors:** Gabriel Oliva, Eder dos Santos, Osiris Sofía, Fernando Umaña, Virginia Massara, Guillermo García Martínez, Cecilia Caruso, German Cariac, Daniela Echevarría, Anabella Fantozzi, Lucas Butti, Donaldo Bran, Juan Gaitán, Daniela Ferrante, Paula Paredes, Erwin Domínguez, Fernando T. Maestre

**Affiliations:** 1 INTA EEA Santa Cruz, Mahatma Gandhi, 1322. 9400 Río Gallegos, Santa Cruz Argentina; 2 INTA EEA, Bariloche, Argentina; 3 INTA Instituto de Suelos, Castelar, Argentina; 4 INTA EEA, Trelew, Argentina; 5 INTA EEA, Esquel, Argentina; 6 INTA EEA, Anguil, Argentina; 7 INTA EEA Valle Inferior de Río Negro – Convenio Provincia de Río Negro – INTA, Viedma, Argentina; 8 Universidad Nacional de la Patagonia Austral, Río Gallegos, Santa Cruz, Argentina; 9 INIA Kampen Aike, Punta Arenas, Chile; 10 Departamento de Ecología, Universidad de Alicante, Carretera de San Vicente del Raspeig s/n, 03690 San Vicente del Raspeig, Alicante Spain; 11 Instituto Multidisciplinar para el Estudio del Medio “Ramón Margalef”, Universidad de Alicante, Carretera de San Vicente del Raspeig s/n, 03690 San Vicente del Raspeig, Alicante Spain

**Keywords:** Ecosystem services, Agroecology, Grassland ecology, Biodiversity

## Abstract

We present the MARAS (Environmental Monitoring of Arid and Semiarid Regions) dataset, which stores vegetation and soil data of 426 rangeland monitoring plots installed throughout Patagonia, a 624.500 km^2^ area of southern Argentina and Chile. Data for each monitoring plot includes basic climatic and landscape features, photographs, 500 point intercepts for vegetation cover, plant species list and biodiversity indexes, 50-m line-intercept transect for vegetation spatial pattern analysis, land function indexes drawn from 11 measures of soil surface characteristics and laboratory soil analysis (pH, conductivity, organic matter, N and texture). Monitoring plots were installed between 2007 and 2019, and are being reassessed at 5-year intervals (247 have been surveyed twice). The MARAS dataset provides a baseline from which to evaluate the impacts of climate change and changes in land use intensity in Patagonian ecosystems, which collectively constitute one of the world´s largest rangeland areas. This dataset will be of interest to scientists exploring key ecological questions such as biodiversity-ecosystem functioning relationships, plant-soil interactions and climatic controls on ecosystem structure and functioning.

## Abstract (summary)

**Table:** 

Measurement(s)	vegetation layer • biodiversity assessment objective • concentration of carbon atom in soil • acidity of soil • concentration of nitrogen atom in soil • structure of soil
Technology Type(s)	quadrat sampling • line intercept sampling • visual observation method • Walkley-Black Method • pH measurement • Kjeldahl method • Bouyoucos method
Factor Type(s)	measurement time & location
Sample Characteristic - Organism	Plantae
Sample Characteristic - Environment	arid biome • arid subtropical
Sample Characteristic - Location	Patagonia

Machine-accessible metadata file describing the reported data: 10.6084/m9.figshare.12761936

## Background & Summary

Drylands, areas where the aridity index - the ratio between precipitation and potential evapotranspiration - is below 0.65, are distributed over 100 of the world’s nations, comprise about 45% of the earth’s land total area^1^, and are highly prone to land degradation and desertification^2^. In addition to expanding the global area covered by drylands, ongoing climate change and the increases in aridity associated with it^3^ are expected to trigger large variations in vegetation cover, biodiversity and key soil properties such as soil carbon content^4^. These ecosystem features largely influence the functioning and capacity of drylands to provide essential ecosystem services, such as biomass production and the maintenance of soil fertility, that sustain the livelihoods of more than 2 billion people living mostly in developing countries^5^.

Programs aiming to describe the status of natural resources and the onset of desertification processes^6^ through change in monitoring plots include variables such as plant cover, species richness and indicators of soil fertility^7^, which are sensitive to changes in environmental conditions and human-induced land degradation^2,8,9^. Additional relevant information is provided by approaches such as the Landscape Function Analysis (LFA)^10^, a field methodology using simple and visual indicators strongly linked to fundamental physical, chemical and biological processes within ecosystems. Monitoring these variables and indicators requires the establishment of a network of field-based observations over regional scales encompassing different ecosystem types. Field data is also needed to calibrate information obtained using remote sensors such as drones and satellite images to use these tools with confidence when monitoring ecosystem change^11^. This task is often done through the cooperation of scientific teams specialized in different ecosystems and working in diverse institutions, and requires the use of common sampling methods to produce comparable information across sites. Examples of regional and global ecosystem monitoring efforts focusing on drylands include West Australia’s WARMS system^12^ the AUS plots-Tern Project^13,14^, the EPES-BIOCOM survey^15^ and the Jornada Monitoring system^16^, which is used by different land management agencies worldwide.

The MARAS (Spanish acronym for “Monitoreo Ambiental de Zonas Áridas y Semiáridas” or “Environmental Monitoring of Arid and Semiarid Regions” in English) network is a field-based ecosystem monitoring protocol developed by the National Institute of Agricultural Technology of Argentina (INTA, https://www.argentina.gob.ar/inta)^17^, which has also been adopted by the National Institute of Agricultural Research of Chile (INIA, http://www.inia.cl/). Development of this methodology started in 2002, with workshops of range scientists backed by the Argentine National Action Against Desertification (PAN, supported by INTA and the German GTZ https://www.argentina.gob.ar/ambiente/bosques/programa-accion-nacional). The initial experimental monitoring plot was set up in the INTA Rio Mayo field experimental station in April 2004, and methodology was improved with further workshops and visits by INTA technicians to the WARMS system^12^ in Perth, Australia (2005) and the Jornada USDA monitoring system^16^ in Las Cruces, New Mexico (2007). An early version of the monitoring system plan was presented in the 2006 Monitoring Science and Technology Symposium organized by the USDA Forest Service in Denver Colorado^18^. The methodology was formalized in an installation manual published by Global Environmental Funds GEF – Programa Naciones Unidas para el Desarrollo UNEP project in 2011^19^. The Chilean INIA team joined the project after receiving field training in 2013 and has thereafter attended the annual meetings that include field work in order to keep methods updated and criteria standardized. Another important milestone of the project was the International workshop that took place in Bariloche in 2014 that gave way to the cooperation with the Laboratorio de Ecología de Zonas Áridas y Cambio Global of the University of Alicante, Spain. Joint Argentinean-Chilean work was intensified after a second international seminar that took place in Punta Arenas (Chile) in 2015.

In Argentina, the MARAS monitoring system is currently applied by six INTA nodes that constitute the national desertification monitoring program. This program received initial funding from the GEF Patagonia Project (UNEP -PNUD ARG 07/G35, 2008–2014), and has subsequently been funded by INTA (“Observatorios de Sustentabilidad Rural” Project, PNNAT-1128035, 2016–19) and the Argentinean Government (Fundación Argentina Proyecto Observatorio, 2015–2016, and Ley 25.422 para la Recuperación de la Ganadería Ovina, 2017–2019). It is currently being supported by INTA (project 2019-PE-E2-I040 “Diseño e implementación de un sistema nacional de monitoreo de la degradación a distintas escalas, con meta en la neutralidad de la degradación de tierras”, 2019–2021). In Chile, data has been contributed by the INIA Kampenaike node and funded by Ministerio de Agricultura through Project 502093-70 (“Sistemas de Praderas Estepáricas de Zonas Frías de Chile”).

**Fig. 1:**
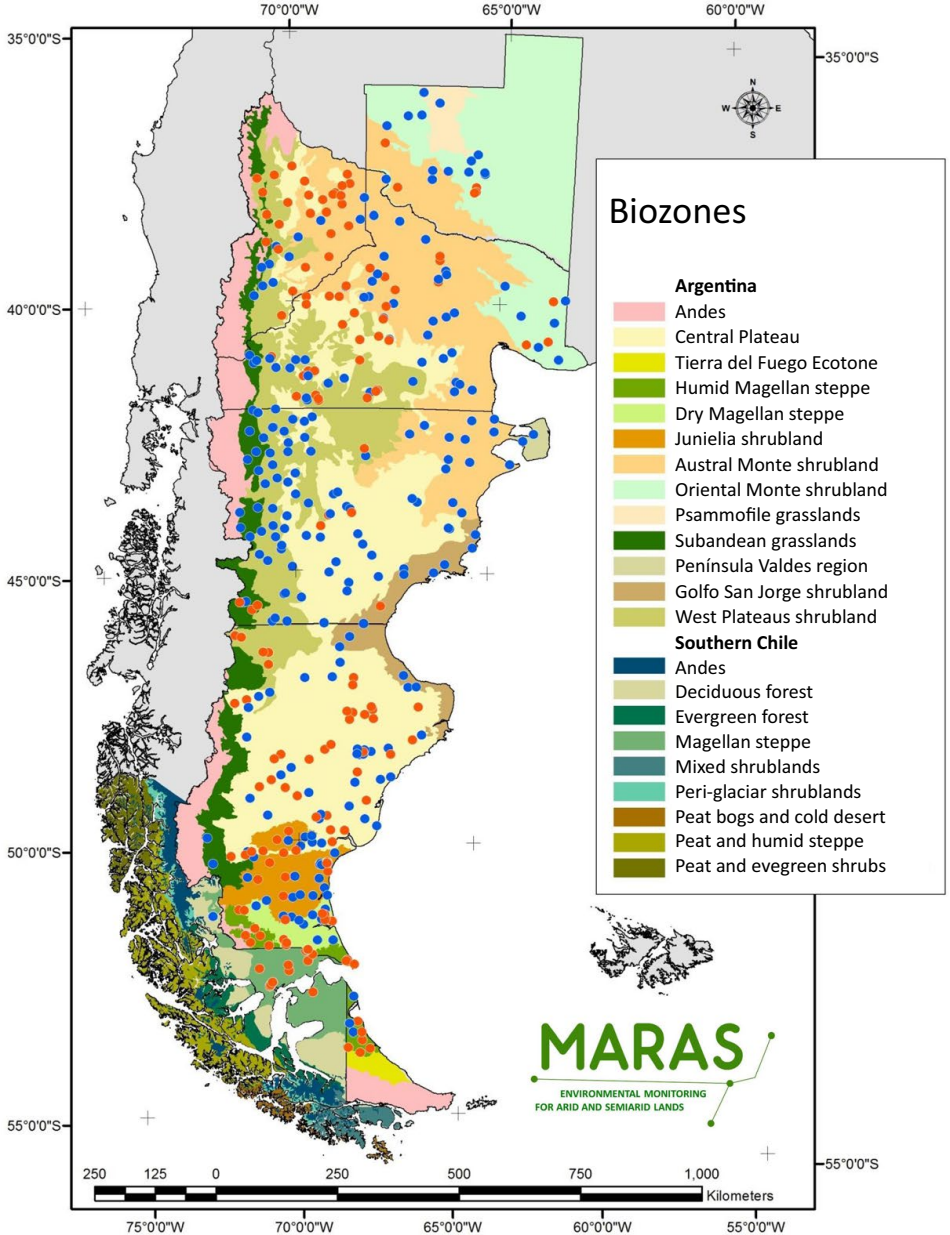
Biozone Map of Argentine and Chilean Patagonia^51,52^ and MARAS monitoring plots installed by February 2020. Red and blue dots have been surveyed only once or at least twice, respectively.

**Fig. 2:**
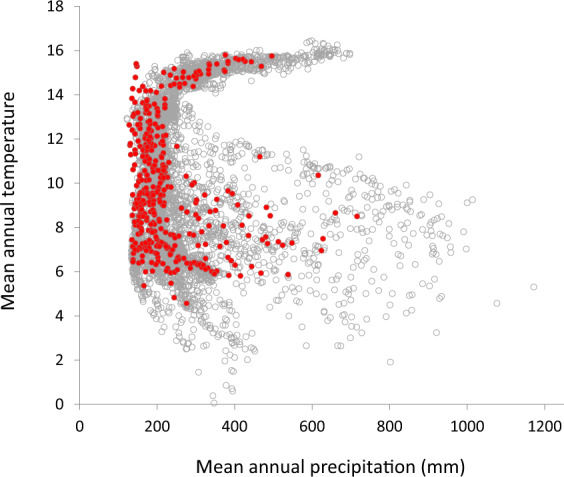
Scatterplot of mean annual temperature and mean annual precipitation of MARAS monitoring plots (red circles) vs. 6000 locations drawn randomly from Patagonia (grey circles). Data obtained from Worldclim https://www.worldclim.org/data/index.html.

Formal setup of the MARAS network of monitoring plots started in 2007, and 426 monitoring plots had been surveyed by December 2019 (Fig. 1, Table 1). They are distributed along a 624.500 km^2^ area that has been classified into 10 biozones^20^, with vegetation ranging from shrublands to grasslands and semi-deserts, and are located under typical grazing regimes present at each site. Monitoring plot locations were selected in order to cover the geographical distribution and habitat heterogeneity of Patagonian biozones. They cover most of the range of climatic space (temperature vs. rainfall) found across Patagonian rangelands (Fig. 2), although extremes of rainfall and low temperatures sites, mostly in Subandean high altitude habitats, are not well represented. A total of 381 farmers voluntarily take part in the MARAS system, most of them with a single monitoring plot. Additionally, 13 monitoring plots were added to fulfill monitoring requirements of mining areas, 6 for public works in dams and 19 for producers that joined sustainable production certifications. Location of these additional monitoring plots follow the main protocol of site selection^21^. Re-evaluation in a 5-year cycle of the 426 monitoring plots is currently in progress, and 247 and 23 monitoring plots have been assessed twice and three times, respectively (Table 2). The protocol used to select monitor plot location in relation to landscape features, ecological sites and a complete description of the techniques used for point-intercept, line intercept transect, Land Function analysis, soil sampling and laboratory analysis can be found in the Installation Manual^21^ and in a previous paper that describes the MARAS system^17^.

**Table 1:** Number of MARAS monitoring plots (Initial assessment) installed in Patagonia by February 10^th^ 2020 tabulated by country and province (Argentina) or region (Chile).

Country and Province	Assessment					Total
	Initial	Second				
	N	N	Years	N	Years	N
Argentina	408	246	5.9	22	8.9	676
Chubut	102	91	5.4	16	9.0	209
La pampa	18	13	7.9	1	9.9	32
Neuquén	39	9	6.1	0	0.0	48
Río Negro	81	50	6.4	1	11.8	132
Santa Cruz	159	79	5.9	4	7.3	242
Tierra del Fuego	9	4	5.5	0	0.0	13
Chile	18	1	1.2	1	3.1	20
Magallanes	15	0	0.0	0	0.0	15
Tierra del Fuego	3	1	1.2	1	3.1	5
Total	426	247	59	23	8.6	696

Second assessment indicates number of monitoring plots that were re-evaluated and the time elapsed in years since the Initial assessment. Third assessment indicates the number of monitoring plots that were re-evaluated a second time and the time elapsed since the initial assessment.

**Table 2:** Number of MARAS monitoring plots installed in Patagonia by February 10^th^ 2020 (Initial assessment) in each Biozone^20^ (regional monitoring unit), number of monitoring plots that were re-evaluated once (Second Assessment), or re-evaluated a second time (Third Assessment) and total number of assessments performed.

Biozones	Assessments			Total
	Initial	Second	Third	
Austral Monte Shrubland	65	34	1	100
Central Plateau	141	74	4	219
Dry Magellan Steppe	25	16	2	43
Golfo San Jorge Shrubland	11	10		21
Humid Magellan Steppe	37	8	1	46
Junellia Shrubland	33	18	1	52
Oriental Monte Shrubland	21	16	1	38
Península Valdez Region	2	2		4
Subandean grasslands	27	19	6	52
West Plateaus Shrublands	64	50	7	121
Total	426	247	23	696

The data presented in this Data Descriptor include

- Location, altitude, stocking density and basic climate variables for 426 monitoring plots.
- Values for 500 sequential point intercepts, each including up to two vascular plant species or type of soil cover (bare soil, rocks, litter, cryptogams) and synthetic variables: total vegetation cover, absolute cover of each species, Richness and Shannon Wiener biodiversity index. Data for 696 plots (426 initial + 247 second + 23 third assessments)
- Initial and final points of sequential patches and interpatches along a 50-m line intercept transect including type of patch (Woody, Herbaceous, Standing dead, Fixed litter) or interpatch (Bare soil, Gravel, Rock, Litter) and width and height of each patch and synthetic variables: mean length of patch/interpatch, mean height of patches, number of patches in 10-m. Data is provided for 680 plots (413 initial + 245 second + 22 third assessments that showed recognizable patch/interpatch structure).
- Initial and final points of 10 interpatches along the 50-m line intercept transect and visual assessment for 11 indicators of soil surface characteristics that act as proxies of soil function. Synthetic variables: Stability, Infiltration/runoff and Nutrient recycling index values for Land Function Analysis^17^. Data included for 677 plots (412 initial + 243 second + 22 third assessments).
- Laboratory analysis (pH, conductivity, organic matter, N and texture) for two composite samples of superficial (1–10 cm) soil, one coming from vegetated patches and the other from interpatch areas devoid of perennial vegetation. Data provided for 397 monitoring plots (295 initial + 87 second + 15 third assessments).

## Methods

Field work was performed by six INTA workgroups based on the cities of Rio Gallegos, Esquel, Trelew, Bariloche, Viedma and Santa Rosa (Argentina), and by one INIA team based on Punta Arenas (Chile). Field crews always included one or more trained botanists, and all of them received joint training and cross-calibration in project workshops that have taken place yearly.

Monitoring plots were chosen to represent the main landscape units of biozones^20^, which were the regional monitoring units, under their usual management: grazed by sheep, cattle or goats, or otherwise free from domestic animals but often grazed by *Lama guanicoe* (guanacos, a wild camelid that is the only large herbivore present in Patagonia^22^). Mean stocking density present at each monitor was established by interviews to the land managers or to local extension officers. It is expressed in Ewe Equivalents (EE), that in south Patagonia is a 49-kg ewe that raises a 20 kg lamb, and consumes about 500 kg of herbage Dry Matter^23^. Patagonian EE is equal to New Zealand’s Ewe Equivalent^24^, 1.51 UGO, the “Unidad ganadera ovina patagonica”, a dry sheep equivalent used in Northern Patagonia^25^, 1.54 Dry sheep units, a similar Australian equivalent^26^. Other domestic livestock, when present, were converted as 1 cow = 6.4 EE, 1 horse = 3.63 EE and 1 goat = 1 EE^27^. Stocking density is also given in the usual cattle equivalent AU Animal Units Year^−1^ that refers to a 454-kg cow that consumes 3200 kg of herbage dry matter^28^ where 1 AU = 0.12 EE.

**Fig. 3:**
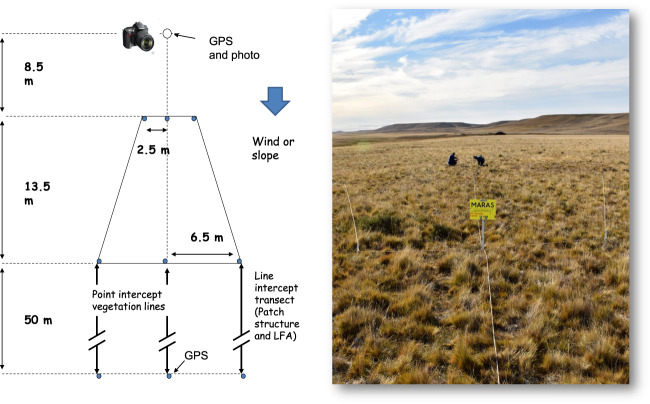
Basic array of the field plots. Fixed steel poles are placed in each point. The photographic plot has a trapezoid shape and is marked with removable ropes. Removable, 50 m -graduated steel tapes are secured between the permanent poles to create two Point-intercept vegetation lines and one Line-intercept transect, which is used for patch/interpatch structure analysis and LFA evaluation. Right: Photo of MARAS TF008 monitoring plot in Tierra del Fuego.

Permanent monitoring plots were placed in uniform areas with a dominant vegetation type and a representative management (typically ewe paddocks in sheep extensive stations), at least 500 m away from water sources and roads. Wetlands or other azonal vegetation types were not sampled. Monitoring plots were geo-referenced using two GPS points and following a standardized method with a single protocol. A manual describing in depth the procedure used to select sites and locate the monitoring plots is available in Spanish^19^ and English^21^ (downloadable from Figshare^29^). Briefly, each monitoring plot consisted in three 50-m transects (Fig. 3) positioned between permanent poles in which vegetation and soil variables were recorded and reassessed every five years. Vegetation was sampled using two 50-m line transects with 250 interception points each, and patch structure was sampled with 50-m line intercept transects for interpatches (areas that lose resources, with a minimum length of 5 cm) and patches (resource sink areas, with a minimum length of 10 cm). Soil stability, infiltration and nutrient cycling were assessed using 11 soil superficial condition indicators recorded in ten bare soil patches (located in interpatch areas) following a modified version of the LFA^30^ methodology adjusted to the cover and litter values found in Patagonia (Table 3). Two 0–10 cm depth composite soil samples were obtained, one from patch and one from interpatch areas, which were analyzed in laboratory for Organic Carbon, N, texture, pH and conductivity. Initially soil samples were discarded, but those obtained in subsequent reassessments have been archived at INTA and INIA node laboratories.

**Table 3:** Modifications introduced to Land Function Analysis (LFA) classes in the MARAS protocol^21^ in relation to the original LFA^30^ manual.

Class	Basal and Canopy cover %		Plant litter cover%		Cryptogam cover%	
	LFA	MARAS	LFA	MARAS	LFA	MARAS
0					0	0
1	<=1	<5	<10	<1	<=1	<1
2	1–10	5–10	10–25	1–10	1–10	1–5
3	10–20	10–20	25–50	10–25	10–50	5–10
4	>20	20–30	50–75	25–50	>50	>10
5		30–40	75–100	>50		
6		40–50				
7		>50				

The MARAS dataset was developed by the Development and Technical Assistance for Third Parties Program (PRODAT) from the National University of Austral Patagonia (UNPA) as part of the ARGENINTA grant (2015–2016), and was initially hosted in the INTA Bariloche servers in 2015. In 2019, within the Ley Ovina 2017–19 grant, the dataset was upgraded to provide a public access viewer https://maras.inta.gob.ar/portal/app/ that generates reports of change in monitoring plots, land units, biozones, provinces and departments and hosting was migrated to the central INTA servers in Buenos Aires. All field data is collected in standardized paper forms (downloadable from Figshare^29^) that are deposited in each node. Information that remained in separate spreadsheet datasets in the initial GEF PNUD 2008–2014 stage was incorporated via data-entry contracts curated by a responsible technician in each node starting in 2015. Available information up to February 10^th^ 2020 was completely transferred to the dataset and is now deposited in Figshare^29^.

### Characteristics of the field survey

MARAS sampling manuals^19,21^ describe the layout of a MARAS monitoring plot, which is similar to that of the WARMS system developed in West Australia^12^. A photographic point (Fig. 3) was fixed and a 72-m central line was laid following the main resource flux direction (wind or water flow). Three poles were fixed at 8.5 m along this line, separated perpendicularly from each other by 2.5 m, and two additional sets of three poles were set at 22 m and 72 m from the photographic point, separated from each other by 6.5 m. Photos were obtained from the photographic pole at 2-m height and from six fixed positions that show a higher detail on the photographic plot and the three lines used to evaluate vegetation and soil. All of them were uploaded to the dataset with a 2000 × 3000-pixel resolution or higher. Positions of pole 1 and 9 were registered with a GPS (Datum WGS84). Three graduated 50-m tapes were placed, two for vegetation surveys and one for patch structure sampling and LFA sampling. All the poles remain *in situ* in order to facilitate the reassessment of the monitoring plots.

**Fig. 4:**
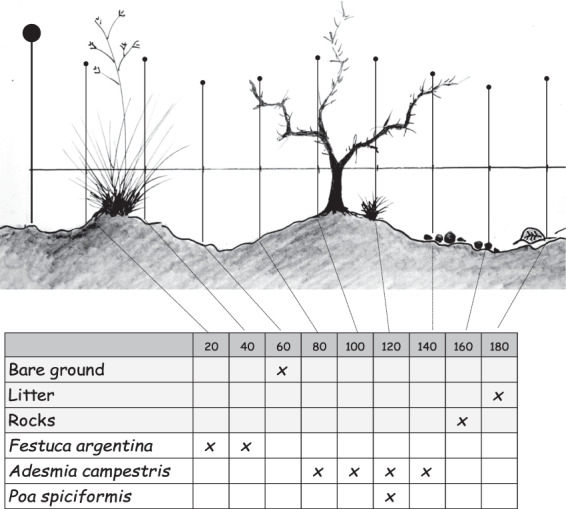
Example of a vegetation point-intercept line. A needle is drawn at 20-cm intervals. All perennial plants striked are identified, and up to two species are recorded, prioritizing the higher strikes. Non-vegetated points are recorded as Bare soil, Litter, Standing dead, Cryptograms, Ephemerals or Rocks. The sequence of strikes is recorded on five 50- point field worksheets in each of the two point-intercept lines.

**Fig. 5:**
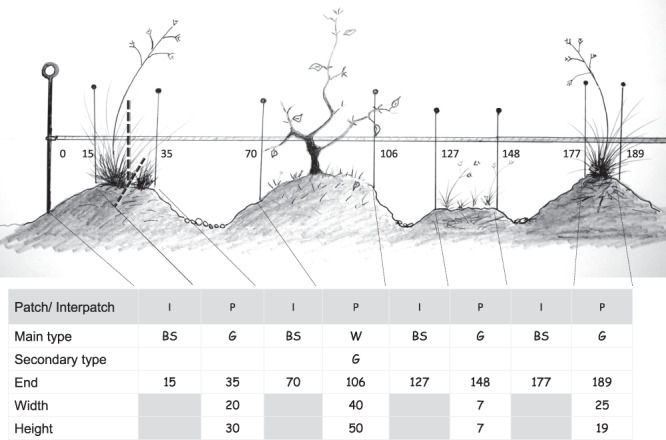
Example of a patch (P)-interpatch (I) sequence in a line-intercept transect. Minimum length for interpatches (areas that lose resources) and patches (areas that retain resources, usually vegetated) is 5 cm and 10 cm, respectively. Main patch types were recorded as herbaceous (G), woody (W) or standing dead (SD). Interpatch types were recorded as bare soil (BS), litter (L), or desert pavement (P). Modal height and width are measured in the center of the patches.

**Fig. 6:**
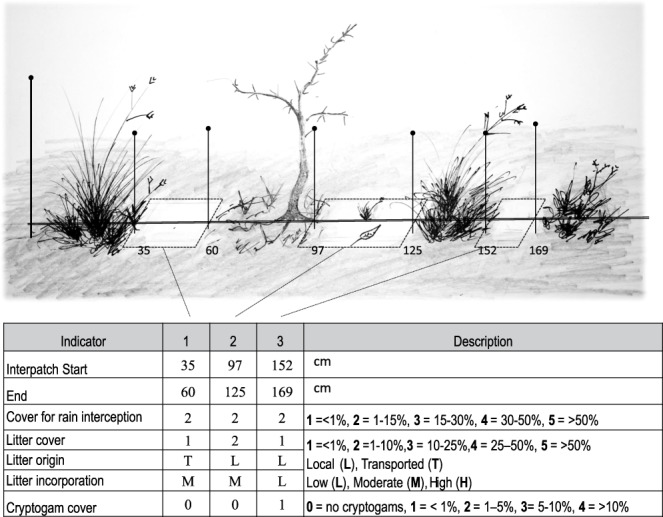
Example of the 20-cm wide and variable length plots placed to measure LFA soil surface indicators. These plots were located on interpatches>40 cm along the line-intercept transect. In ten of these plots, 11 soil, type of vegetation and litter cover indicators were recorded to estimate Infiltration, Nutrient Recycling and Stability indices according to the Landscape Function Analysis^30^ methodology. The sample worksheet shows how the first three plots and some indicators are recorded.

Four basic field methods to estimate vegetation and soil properties were used at each monitoring plot:

(1) Point intercept lines, with 500 sequential point intercepts^31^ at 20-cm intervals along two 50 m graduated lines (Fig. 4). At each point, a needle was set and perennial vascular plants were identified. In case of foliage superposition, the two higher plants were recorded. Plants that could not be recognized in the field were collected and voucher specimens were classified in the laboratory. All plants were identified to the level of species or pseudo species (identified to genus). Once the point intercept lines were finished, a thorough visual search was made along the central point intercept line including the photographic plot and additional plant species were listed. Starting in 2015 this was done following the point and flexible area methodology^32^. The number of individuals and approximate dimensions (length × width) of new species within 1 m at each side of the central transect were recorded. Plants that were farther away were recorded as number, dimensions and modal distance from the central transect. These data are not yet available in the dataset. A total of 636 vascular plants^33^ have been identified across all MARAS monitoring plots surveyed so far. Values for cover of each species, Rocks, Bare soil, Litter, Ephemerals, Standing dead, Cryptogams were estimated as number of strikes on each category in the point intercept line divided by the total number of intercept points (500), and are expressed in %. Total vegetation cover cannot be estimated by sum of cover of each species because double vegetation strikes are accepted in the point intercept. It was estimated instead as the complement of non-vegetated point intercepts: 100- (Bare soil % + Rock % + Litter % + Standing dead% + Cryptogam %). This data is available for all the monitoring plots.

(2) In drylands, some portions of the landscape are enriched by trapping resources that are transferred from bare ground areas devoid of perennial vegetation (“interpatches”) to sink areas or “patches” (typically discrete perennial vegetation and/or litter/wood accumulation patches^34^). Interpatches are therefore nutrient-poor areas where resources (soil, propagules, nutrients) are lost, while patches are nutrient-rich areas that retain them, a pattern that takes place at different scales. In the MARAS protocol they are evaluated at site-scale (patterns generated by accumulation by shrubs or grasses) using line intercept transects that record patch structure along the soil 50-m line. The criteria used in order to define patches and interpatches is detailed in the MARAS manual^21^. The length of successive patches (minimum length 10 cm) and interpatches (minimum length 5 cm) was noted in each transect. All patches were classified as herbaceous, woody or standing dead. The height and width of each patch, measured perpendicularly at the middle point of each patch with a ruler, were also recorded. Interpatches were classified as bare soil, litter, or desert pavement (Fig. 5). This data is available for 681 plots (413 initial, 246 second and 22 third assessments) that showed patch/interpatch structure.

(3) Soil surface condition was assessed using the LFA^30^ methodology along the line-intercept transect (Fig. 6). Plots were outlined over the first ten interpatches that exceeded 40 cm in length, a modification of the original methodology^10,30^ where plots are systematically placed at intervals along a line. At each sampling point, which was 20-cm wide and had variable length, eleven indicators of soil surface conditions were visually estimated: (1) Aerial cover for rain interception, (2) Basal cover of patches, (3) Litter cover, origin and degree of incorporation, (4) Cryptogram cover, (5) Erosion features: hummocks, desert pavements, microrills or rills, (6) Deposited materials, (7) Microtopography, (8) Soil crust type and degree to which it is disturbed, (9) Surface crust resistance, (10) Slake test: time that soil aggregates retain integrity in water and (11) Texture. We adapted the range of indicators in each class of the original LFA methodology^30^ to the soil and vegetation characteristics of Patagonia (Table 3). The sum of ratings obtained with these indicators for a particular monitoring plot was divided by the sum of the maximum values of the indicators (that represents 100%) and expressed as percentage. Three LFA indexes were estimated based on combinations of these indicators: Stability, Infiltration and Nutrient Cycling, which were calculated accordingly to the MARAS manual^21^. Data is provided for the 677 plots (412 initial, 243 second and 22 third assessments) that showed interpatches.

(4) Two composite 0–10 cm depth soil samples (consisting on five subsamples in patches and five in interpatches) were collected in patches (areas that accumulate resources, usually vegetated) and interpatches (areas that lose resources, usually bare soil). These samples were analyzed in the laboratory for pH 1:2.5, conductivity (dS/m), Organic matter following the Walkley - Black^35^ method (%), organic carbon, approximately 58% of organic matter, was estimated as organic matter/1,724^36^, N content by modified Kjeldahl procedure (%) and texture was obtained using a using Bouyoucos hydrometer. This data is available for 397 monitoring plots (295 initial + 87 second + 15 third assessments).

## Dataset

The main MARAS database was built using open source software tools PHP 5.3 (www.php.net) and PostgreSQL (www.postgresql.org). The source code has a GNU General Public License (GPL), and is hosted in the main INTA servers https://maras.inta.gob.ar/app/. It is accessible with passwords, but a public viewer is available at https://maras.inta.gob.ar/. This free browser delivers maps, photographs, basic monitoring plot information and change reports at monitoring plot and regional scales. It is currently available only in Spanish, but automatic translation to English is acceptable in most web browsers. It includes a GIS map-based walkthrough with image layers of Open Street Map (https://www.openstreetmap.org). The MARAS dataset is frequently updated through six nodes in Argentina and one in Chile, and the database program code also receives upgrades. A complete backup of the information, code and images of the dataset updated to February 2020 has been deposited in Figshare^29^. This dataset will be updated annually in Figshare to reflect data additions and updates to the code.

## Data Records

The dataset presented in Figshare^29^ is a copy of the dataset as it was on 10^th^ February 2020. By this date, a total number of 426 monitoring plots were setup in Patagonia. A single, initial assessment is presented for 156 monitoring plots, 247 of them also show data of a second assessment after a mean period of 5.9 years and 23 of them have a third, additional assessment after a mean period of 8.6 years since their set up (Table 1). A total number of 696 monitoring plot readings are in this way available in the MARAS Figshare files. Monitoring plots are distributed in six provinces of Argentina and two provinces (or regions) of Chile (Table 1), and political division of land also includes 52 departments. Biophysical classification includes 12 Biozones^20^ (Table 2) and 35 Landscape units^37^. The dataset stores additional information on the 381 farms including owners or traditional landholders, and managers. Stocking data is provided in the.csv and.xls files.

“Google earth file of MARAS monitoring plots” is a.kml file showing the location of MARAS monitoring plots. Information includes Country, Province, Location number, Site Name, Date, Long, Lat, Altitude SRTM_30 (m.a.s.l), Department, Landform, Biozone, Mean annual temperature (in °C, obtained from Worldclim) and Mean annual rainfall (mm, obtained from Worldclim^38^ https://www.worldclim.org/data/index.html).

“MARAS monitoring plots csv format” is a.csv file, with a similar file available in.xls format that present the location and main vegetation, soil, land function and floristic variables for 426 MARAS monitoring plots, including 247 second assessments and 23 third assessments. Variables of this file are described in Online only Table 1. Each one of the 687 rows represents a monitoring plot reading with location, name, date of assessment, mean temperature and annual precipitation, aridity index and stocking rate data. Vegetation variables are cover, diversity, bare soil, ephemerals, standing dead and cryptogams. Vegetation structure includes patch and interpatch length, basal cover, patch height and width. Land Function Analysis includes indexes of Stability, Infiltration and Nutrient recycling. Soil laboratory results for two samples (patch and interpatch) include pH 1:2.5, conductivity (dS/m), Organic matter (%), organic carbon, Nitrogen (%), clay, silt and sand content (%). The rest of the columns are absolute cover values for all the vascular perennial species detected. Nomenclature follows “Flora del Cono Sur”^33^. Note that the sum of all absolute species cover can be >100% given that the species may be superimposed in two strata (up to two species strikes are recorded per point).

“MARAS SQL Database” is a.sql file that contains the dataset information, which includes the sequence of strikes per species in the line transects, the sequence of patches and interpatches and the individual indicator values for the LFA plots, as well as other information such as sheep or cattle numbers. This information is accessible through the MARAS program that can be restored and installed in a server via the maras-files.tgz file or browsed using other SQL software. It requires PostgreSQL.

“MARAS program and photographs” is a 12.3 Gb compressed.tgz file that upon restoration generates a complete version of the system written in PHP 5.3 (www.php.net) and PostgreSQL (www.postgresql.org) program code. In order to access the database a password must be set up in the file/exports/production/maras-www/bd/Bd.Main.Configuraciones.Class.php. Additionally, the .tgz file will generate a photograph file folder tree with the structure: /exports/production/maras-www/imagenes/fotos_monitores/id.monitoring_plot/id.observation. The “id.monitoring plot” folders are code numbers of each MARAS monitoring plot, as listed in Online only Table 1. The “id.observation” folder is the number of assessment of the monitoring plot. Each “id.observation” folder has seven files: /diag.der; /diag.izq; /foto-grupo; /poste_central; /transecta_central; /transecta_lateral; /transecta_suelo. These files store the photographs of monitoring plots and observation teams in jpg format, with the original resolution and taken from different positions as detailed in the MARAS manual^21^. For most ecological applications it is not necessary to interrogate the SQL database or restore the compressed files, as the location of monitoring plots including climatic data, stocking density and main vegetation, patch structure, land function, soil analysis and vascular plant species cover have been summarized together in the .csv and .xls files “MARAS monitoring plots”. More intensive geostatistical uses may require information on the sequence of point intercepts or patches, and this can be recovered from the SQL database directly or by means of the restored database program.

“Manual de monitores MARAS” is a Spanish (original) version of the installation manual^19^ in .pdf format. “MARAS manual English version” is an English version of the MARAS installation manual^21^ in .pdf format. “MARAS field worksheets_June 2020” and “Planillas de campo MARAS version 2020” are .pdf files with the worksheets used during field data recording. “Shapefile_Scidata” is a .zip file with Administrative units, biozones and land units used in shape format.

## Technical Validation

### Errors associated to MARAS estimations

The MARAS system uses a single methodology and sampling effort (500 transect points along two 50-m lines, 50-m line- intercept transects and 10 LFA plots) to estimate cover, patch-interpatch structure and LFA indicators over a wide range of vegetation types, from shrublands to grasslands or semi deserts. Whether the estimations are representative of the real mean value of the monitoring plot or not depends heavily on the grain of the vegetation heterogeneity in relation to the length and resolution of sampling lines. We assessed errors associated with these estimations using the minimum number of samples equation^39^:

1 $${\rm{n}}=\frac{{({Z}_{\alpha /2})}^{2}{(\sigma )}^{2}}{{{\rm{E}}}^{2}}$$

where

n = number of samples

Z_α/2_ = False-change Type I error rate

σ = Standard deviation

E = Error in absolute terms.

Where σ (intra plot standard deviation) was estimated in 5 monitors randomly selected for each biozone. The standard deviation for point intercepts was obtained dividing 50-m lines in 10 subsamples with 50 points each (n = 10). In the case of interpatch length (n = 25 to 50 interpatch length measures) and LFA plots (n = 10 measures), standard deviation was estimated directly. False-change Type I error rate Z_α/2_ was set at 1.96 (0.05 probability). Given that at the plot scale the sampling effort is fixed (500 points, 25–50 patch-interpatch pairs and 10 LFA plots), this equation was modified to estimate the error.

2 $${\rm{E}}=\sqrt{\frac{{({Z}_{\alpha /2})}^{2}{(\sigma )}^{2}}{{\rm{N}}}}$$

Errors associated to monitoring plot estimations using Equation 2 are shown in Table 4. Lines with 500 intercept points estimate plot total vegetation cover within an error of 4.5%. The line intercept transects consisting of at least 50 patch-interpatch pairs provided interpatch length estimations within a 26 cm error, and the 10 LFA Stability index observations provided estimation errors within 4 units. However, coarse-grained vegetation types such as Austral Monte Shrublands have a higher error (5.8%, 71 cm and 4.2 units, respectively).

**Table 4:** Errors associated to site means estimations using the prescribed sampling effort of MARAS (500 intercept points, 50 patch-interpatch pairs in line-intercept transects and 10 LFA plots) estimated from five monitoring plots in each of the main Biozones of Patagonia using Equation 1 (n/a= errors not assessed).

Biozones	Cover	Interpatch length	Stability index
	% absolute cover	cm	LFA units
Austral Monte Shrubland	5.8	71	4.2
Central Plateau	5.1	20	3.3
Dry Magellan Steppe	4.2	10	4.0
Golfo San Jorge Shrubland	5.3	25	4.2
Humid Magellan Steppe	2.8	27	4.1
Junellia Shrubland	6.3	10	3.8
Oriental Monte Shrubland	n/a	n/a	n/a
Península Valdez Region	n/a	n/a	n/a
Subandean grasslands	4.1	15	3.8
West Plateaus Shrublands	2.3	28	3.4
Total	4.5	26	3.8

This error analysis could not be applied to diversity estimations at the monitoring plot scale, as it is well known that the number of species in a point intercept line does not stabilize with increasing sampling effort, as new, rare species keep appearing with added point intercepts^40^. The number of species detected with the MARAS 500-point line protocol is therefore a sub estimation of total plant biodiversity. To evaluate the precision of the protocol´s estimation of richness as a proxy of monitoring plot biodiversity we analyzed a subset of 160 monitoring plots, where species richness obtained in the MARAS monitoring plots (MR: species detected in the 500 points) was correlated with the total number of species identified by a dedicated and thorough visual search of the whole MARAS monitoring plot including the photographic plot and the area between the 50 m transects (R: species detected by thorough inspection). The linear regression of total species count with the number of species detected by point intercept (y = 1,03x + 5,77) has an R² = 0,8154 (P < 0.01)^17^. In this way, although the species count of MARAS lines underestimated total richness (and missed approximately 6 species in each monitoring plot), the high R² value and a slope close to 1 indicates that it is an effective estimator of the α-diversity of each plot.

### Minimum sample size for biozones

Inter-plot differences arise within the biozones surveyed due to climatic or soil heterogeneity, and to differences in grazing management. The minimum sampling effort is the number of monitors necessary to achieve a single estimation within an acceptable error^16^. It was estimated using Equation 1 by analyzing the standard deviation of four main variables between monitoring plots at each biozone. Sample size estimation at regional scale (Table 5) indicates that the 426 monitoring plots installed by December 2019 were enough to estimate the vegetation cover, species richness and LFA mean within the 10% error target (equivalent to ±5% cover, ±2 species of richness, and ±5 LFA units of the general mean). Not enough monitors were in place to estimate Interpatch length within 10% error (±12 cm), but it nevertheless provides an estimation of this variable with a 15% error (±19 cm). This analysis holds for most biozones, where the existing number of monitoring plots are enough to satisfy the 10% and 15% error targets, except for some heterogeneous biozones such as Subandean grasslands and Golfo San Jorge shrublands, where additional monitoring plots should be established to satisfy them. The underlying problem in some Biozones such as the Subandean grasslands may be that they encompass too much environmental heterogeneity and should be subdivided further to adequately detect changes in the future. The limitations of the analysis of vegetation structure in high cover areas through line intercept transects becomes evident in the Humid Magellan Steppe, where interpatches are few and mostly associated to walking trails of domestic stock. In this conditions Interpatch length (and its complementary variable Patch length) varies substantially and cannot be evaluated with an acceptable error. We have kept the line intercept transects mandatory for these habitats in the prospect that a recognizable patch/interpatch structure may arise with degradation processes in the future.

**Table 5:** Number of monitoring plots installed by December 2019, and minimum sample in order to estimate the mean of each Biozone within a 10% error of the mean for Vegetation cover, species richness and LFA Stability Index and within a 15% error for Interpatch length using Equation 2.

Biozones	Monitoring plots installed	Minimum number of monitoring plots required			
		Vegetation cover	Species Richness	Interpatch length	LFA Stability index
Austral Monte Shrubland	65	50	43	28	28
Central Plateau	141	35	42	120	16
Dry Magellan Steppe	25	12	13	24	11
Golfo San Jorge shrubland	11	37	23	42	14
Humid Magellan Steppe	37	7	16	89	29
Junellia shrubland	33	9	36	16	8
Oriental Monte Shrubland	21	n/a	n/a	n/a	n/a
Peninsula Valdez	2	n/a	n/a	n/a	n/a
Subandean grasslands	27	36	96	62	17
West Plateaus shrublands	64	41	47	47	19
Total	426	227	316	428	142

### Repeated measures

Changes in vegetation and soil induced by climate, management or natural events can be tracked with descriptive statistics and maps, but the system should be able to detect the statistical significance of these changes at regional and Biozone scales. The power to detect change depends on the number of monitoring plots deployed by region or Biozone, but also on the observational errors, which are inevitable and variable according to the techniques applied. In the MARAS sampling design, plots and Biozones are not sampled randomly each time, as sites are re-localized and measuring tapes set between poles permanently fixed in the monitoring plots. Even in this case, errors arise due to random factors such as misalignment of reading tapes and because observers are likely to change, and each have their own skills, criteria and biases. Monitoring plots may be treated in this experimental design as individuals in paired T-tests or repeated-measures ANOVA, and the number of monitors necessary to detect change with a given error in consecutive estimations may be estimated from the standard deviation of the differences between samples as follows^39^:

3 $$\frac{{({\rm{sdiff}})}^{2}{({\rm{Z}}\alpha +{\rm{Z}}\beta )}^{2}}{{{\rm{MDC}}}^{2}}$$

where:

sdiff = Standard deviation of the differences between paired samples

Z_α_ = Z-coefficient for the false-change (Type I) error rate

Z_β_ = Z-coefficient for the missed-change (Type II) error rate.

MDC = Minimum detectable change size in absolute terms.

Observational errors in repeated estimations using the MARAS protocol were estimated using a set of monitoring plots that were assessed by different teams of technicians yearly from 2012 to 2015. This is not the usual 5-year assessment interval and was done in order to fulfill particular monitoring requirements of the Vanguardia Mining site 48° 20′ 30.7′′ S; 68° 8′ 50.6′′ W. These eight monitoring plots were set on desertic perennial dwarf shrublands of the Central Plateaus, and located in a 10-km radius with similar topographic positions and no grazing. In these conditions climatic and management differences between monitoring plots were minimal and vegetation showed small changes and, most importantly, similar interannual trends. We assumed that in these conditions most of the variability in change estimation between plots (Sdiff) was attributable to observational errors of three different evaluation teams that performed the assessments. The variability of the differences was used therefore to estimate MDC at monitoring unit scale in Equation 3. In this analysis Z_α_ and Z_β_ were set at 1.96 and 1.64 respectively (5% error rate).

Paired sample interannual differences (Diff) between monitoring plots in the Vanguardia site between 2012 and 2015 (Table 6) were small: +/−0.2% of vegetation cover, +/−0.4 species richness, +/−7 cm of interpatch length and +/−2.6 units of LFA stability index. Standard deviation of these difference values (Sdiff) were 3–10 times higher in all cases, indicating that annual variation in monitoring plots was lower than the observational errors due to imprecision and bias of the observers, especially in the interpatch and stability LFA index measures, which showed yearly variations of 20 cm and 9 units respectively. Minimum detectable change analysis showed that a set of 10 monitoring plots would be able to detect small changes in cover (2.2%) and species richness (1.8 species), but increased observational variability in readings of Interpatch size and Stability LFA index reduce the power of change detection to relatively high levels of variation of 23 cm and 10% LFA units in consecutive readings (Table 6).

**Table 6:** Yearly paired-sample mean differences and standard deviation of the differences (Sdiff) for vegetation cover, species richness, interpatch length and LFA stability index for five monitoring plots of the Vanguardia site in Santa Cruz. MDC (Minimum detectable change) using a sample of n=10 or 5 monitors to estimate change using Equation 2.

Year	Vegetation cover (abs cover %)		Species Richness (n° of species)		Interpatch length (cm)		LFA Stability Index (LFA units)	
	Diff	Sdiff	Diff	Sdiff	Diff	Sdiff	Diff	Sdiff
2012–2013	−0.3	2.02	−0.2	1.10	−11.1	25.85	1.1	5.44
2013–2014	0.2	1.62	−0.6	1.14	7.1	18.97	−3.5	4.61
2014–2015	−0.2	2.51	0.5	2.30	−3.3	14.14	−3.3	10.73
Mean +/-	0.2	2.00	0.4	1.58	7.1	20.42	2.6	8.68
Sample size	MDC		MDC		MDC		MDC	
10 monitoring plots	2.2%		1.8 species		23 cm		10 LFA units	
5 monitoring plots	3.2%		2.5 species		33 cm		14 LFA units	

### Possible use of this data

The MARAS data allow for evaluations of ecosystem change with a precision that has not been previously possible at the regional scale in South America. The MARAS monitoring plots are thus useful for a wide variety of floristic, ecological and biogeographic studies, and have been used to describe biodiversity patterns in Patagonia^41^, to quantify the relative importance of biotic and abiotic factors as drivers of regional variations in plant productivity^42^ and soil organic carbon^4^, to validate information from satellite data on the ground^43^, to assess how biodiversity modulates ecosystem responses to drought^44^ and to explore how aridity and overgrazing affect the structure and functioning of drylands^45^. From the land manager perspective, the MARAS system has been used to compare the effects of different grazing systems^46,47^ and in certification schemes such as sustainable management of grasslands^48^, Responsible Wool^49^ or Organic Production. MARAS is also being used to monitor vegetation restoration programs set up by the gold mining industry and to assess the effects of a large-scale hydroelectric project on the vegetation of the Santa Cruz river basin http://represaspatagonia.com.ar/index.php/en/home.

The data provided by MARAS are particularly helpful to interpret changes in “slow” variables^6^ that have lengthy turnover times and are related to ecosystem attributes linked to climate change and variations in land use intensity, two key components of on-going global environmental change. They also provide relevant data to fulfill monitoring requirements of UN convention to Combat Desertification (UNCCD) and related conventions, the United Nations Framework Convention on Climate Change (UNFCCC) and the Convention on Biological Diversity (CBD). Therefore, the MARAS data is of interest to scientists, administrators and land managers alike, as well as to those aiming to setup ecosystem monitoring programs in drylands worldwide.

### Future directions

Most of the future effort of the MARAS project will be invested in completing the re-sampling of established plots in Patagonia and keeping the dataset and the international repository updated. There will be a decreasing emphasis on new plots over time, and the methodology will be promoted as a reliable way to certify sustainable management within extensive grazing production protocols (a way to densify the network of monitoring plots and to cover some of the costs of maintaining the MARAS system). Work will be directed towards securing the long-time funding of the program and expanding the spatial coverage of key Argentine and Chilean arid and semi-arid regions, including Puna and Monte, and integrating it with complimentary monitoring systems developed for the xeric woodlands of Chaco and Espinal^50^. This will require new monitoring nodes, probably based on agreements with other institutions from Argentina and Chile.

## Usage Notes

When using data from MARAS dataset please cite this publication. Both data and database code are available under a Creative Commons Attribution 4.0 International Public License, whereby anyone may freely use data and adapt our database, as long as the original source is credited, the original license is linked, and any changes to our data are indicated in subsequent use.

## Data Availability

The code of the MARAS database was designed in SQL and written in Postgre SQL. It is being periodically upgraded but the latest version (10^th^ February 2020) is freely available through the “maras-files.tgz” file in figshare^29^. Future versions will be uploaded to this dataset on an annual basis.

## Online-only Table

**Online-only Table 1:** Description of variables included in the Figshare “Maras monitoring plots” file and example of data included in the SC001 monitoring plot.

Variable	Description	Example
Country	Country of monitoring plot	Argentina
Province	Province of monitoring plot	Santa Cruz
Database_monitor_code	Code of the monitoring plot in the database	19
MARAS_number	Monitoring plot number	#SC-001
Site_Name	Name of the farm or site of installation	Potrok Aike
Date	Date of set up/ reevaluation of monitoring plot	17/10/2008
Assessment	Number of successive observations of this location 1= initial 2=first reassessment 3=second reassessment	1
Period_days	Days from the initial observation to the current assessment (initial=0)	0
Lat	Location of photographic pole (deg° min’ sec.dec” lat S) Datum Wgs84	51°55'21.6“S
Long	Location of photographic pole (deg° min’ sec.dec” long W) Datum Wgs84	70°25'35.8“W
Google_map_link	Google Earth hyperlink to location	Link
Altitude_SRTM30_m	Elevation (meters above sea level) from SRTM_30	120
Stocking density_EE_ha_yr		0.267
Stocking density_AU_ha_yr	Domestic stock density in Ewe equivalents^30^ EE =500 kg Dry matter consumption	0.042
Aridity_index_v2	Global Aridity Index values v2: Aridity Index (AI) = MAP / MAE Where: MAP = Mean Annual Precipitation and MAE = Mean Annual Potential Evapotranspirationhttps://cgiarcsi.community/data/global-aridity-and-pet-database/	0.252
Departament	Department (Political division)	Güer Aike
Landform	Landform according to Lopez et al. 2005 map^37^	Glacial flatland
Biozone	Biozone according to Bran et al. 2005 map^20^	Dry Magellan Steppe
MAP_mean_mm	Mean annual precipitation from Worlclimhttps://www.worldclim.org/data/index.html	200
Temp_mean_C	Mean annual temperature Worldclim https://www.worldclim.org/data/index.html	6.8
Vegetation_cover_%	Vegetation cover in point intercept line: 100- (bare soil % + rock % + litter % + standing dead% + cryptogam %)	63
Richness_n_species	Number of species present at least in one point in the point quadrat line	26
Shannon-Wiener	H´= ∑ p1* ln (pi) Where pi is relative cover of species i estimated as pi= number of points species 1 / total number of vegetation points.	2.16
Rocks or stones_%	Number of strikes on rocks or stones>2 cm diameter in the point intercept line /500 in %	0
Bare soil_%	Number of bare soil strikes in the point intercept line /500 in %	15.8
Litter_%	Number of litter (dead plant material not attached to the plant) strikes in the point intercept line /500 in %	4.8
Ephemerals_%	Number of Ephemeral (annual plant) strikes in the point intercept line /500 in %	5.6
Standing dead_%	Number of standing dead (dead plant material still attached to mother plant) strikes in the point intercept line /500 in %	4.8
Cryptogams_%	Number of cryptogam (non-vascular plants including mosses and lichens) strikes in the point intercept line /500	6
Patch_basal_cover_%	Sum of the longitudes of patches along the intercept line/ 5000 cm×100 (%)	64.51
Interpatch_length_cm	Sum of longitudes of interpatches / number of interpatches (cm)	39.4
Patch_length_cm	Mean length of patches (cm)	70.1
Patch_width_cm	Sum of width of patches / number of patches (cm)	NA
Patch_height_cm	Sum of height of patches / number of patches (cm)	4.7
Number_patches_10m	Number of patches in the transect /5	9.2
Stability_LFA_index	The ability of soil to resist erosive forces. It is the sum of the indicators: Aerial soil cover (1–5) + Litter cover (1–5) + Cryptogam cover (0–4) + Soil erosion (1–4) + Deposited materials (1–4) + Presence/integrity of crusts (1–4) + Soil surface resistance (1–4) + Slake test (0–4) divided by maximum score (34) in %	60.88
Infiltration_LFA_index	An estimate of proportion of rainfall that infiltrates the soil (and is therefore available for plants) in relation to superficial runoff. Calculated as the sum of scores: Basal cover of patches (1–7) + Micro topography (1–5) + Slake test (0–4) + Litter (cover, origin, incorporation) (1–20*) + Soil surface resistance (1–10**) + Texture (1–4) divided by the maximum score (50) in %. *Litter contributes to this index by multiplying the cover class value by the coefficients: Transported (T)=1, Local (L)=1.5, Low incorporation (L)=1, Moderate incorporation (M)=1.7, High incorporation (H)=2. Example: An LFA plot that has 10–25% litter, of local origin and moderately incorporated (registered as 2LM) would contribute 2×1.5×1.7=5.1 ** Infiltration is reduced in compact surfaces. In this index the original values for surface resistance were recoded Class=4 recoded 1, Class 3 recoded 3.3, Class 2 recoded 6.6, Class 1 recoded 10.	51.63
Nutrient_recycling_LFA_index	Relates to the rate of decomposition of organic matter that enables nutrients to recycle in the soil. Calculated as the sum of scores for: Basal cover of patches (1–7) + Micro topography (1–5) + Cryptogam cover (0–4) + Litter (cover, origin, incorporation) (1–20*) *Litter contribution to this index is computed in the same way as the previous infiltration/runoff index.	39.,37
Patch_lab_number	Number of laboratory soil sample	65087
Patch_conductivity_dS/m	Conductivity of soil sample (dS/m)	0.14
Patch_pH	pH: 1:2.5 (LS-PT-xx)	6.4
Patch_Organic_Carbon_%	Organic carbon content (organic matter/1,724)	1.13
Patch_Nitrogen_Kjeldahl_%	N content by modified Kjeldahl (LS-PT-13) in %	0.13
Patch_Organic_matter_%	Organic matter by Walkley - Black (LS-PT-06) in %	1.95
Patch_clay_<2μ_%	Patch Clay (% m/m)	7.5
Patch_silt_2–50μ_%	Loam content (% m/m) 2–50μ Bouyoucos hydrometer (LS-PT-03)	11.2
Patch_sand_50–2000μ_%	Sand content (% m/m) 50–2000μ Bouyoucos hydrometer (LS-PT-03)	81.4
Interpatch_lab_number	Number of laboratory soil sample	65201
Interpatch_conductivity_dS/m	Conductivity of soil sample (dS/m)	0.33
Interpatch_pH	pH: 1:2,5	6.29
Interpatch_Organic_Carbon_%	Organic carbon content (organic matter/1,724)	1.22
Interpatch_Nitrogen_Kjeldahl_%	N content Kjeldahl modified (LS-PT-13)	0.1
Interpatch_Organic_matter_%	Organic matter Walkley - Black (LS-PT-06)	2.1
Interpatch_clay_<2μ_%	Clay content (% m/m) < 2μ Bouyoucos hydrometer (LS-PT-03)	7.5
Interpatch_silt_2–50μ_%	Silt content (% m/m) 2–50μ Bouyoucos hydrometer (LS-PT-03)	14
Interpatch_sand_50–2000μ_%	Sand content (% m/m) 50–2000μ Bouyoucos hydrometer (LS-PT-03)	77.8
